# Magnesium batteries: Current state of the art, issues and future perspectives

**DOI:** 10.3762/bjnano.5.143

**Published:** 2014-08-18

**Authors:** Rana Mohtadi, Fuminori Mizuno

**Affiliations:** 1 Materials Research Department, Toyota Research Institute of North America, Ann Arbor MI 48105 (USA), Fax: (+1)734-995-2549

**Keywords:** cathode, electrolyte, magnesium anode, magnesium battery, magnesium metal

## Abstract

*“...each metal has a certain power, which is different from metal to metal, of setting the electric fluid in motion...”* Count Alessandro Volta. Inspired by the first rechargeable magnesium battery prototype at the dawn of the 21st century, several research groups have embarked on a quest to realize its full potential. Despite the technical accomplishments made thus far, challenges, on the material level, hamper the realization of a practical rechargeable magnesium battery. These are marked by the absence of practical cathodes, appropriate electrolytes and extremely sluggish reaction kinetics. Over the past few years, an increased interest in this technology has resulted in new promising materials and innovative approaches aiming to overcome the existing hurdles. Nonetheless, the current challenges call for further dedicated research efforts encompassing fundamental understanding of the core components and how they interact with each other to offering new innovative solutions. In this review, we seek to highlight the most recent developments made and offer our perspectives on how to overcome some of the remaining challenges.

## Introduction

Fueled by an ever increasing demand for electrical energy to power the numerous aspects of modern human life, energy storage systems or batteries occupy a central role in driving the electrification of our societies [1]. The basic principles of a battery are rather old; its invention by Allessandro Volta dates back to the eighteenth century [2] (archeological findings in the 20th century even suggest that the first battery was developed in Mesopotamia dating back to 2000 BC, to what is referred to as the “Baghdad battery” [3]). Since its invention, and most particularly in the twentieth century, advancements in energy storage technologies continued to evolve over time resulting in a myriad of distinct batteries and energy storage chemistries [1]. Out of the several known battery technologies, secondary or rechargeable batteries, such as nickel metal hydride and lithium-ion, which allow for reversibly storing and harnessing power on demand while providing high power and energy conversion efficiencies, have played an invaluable role in driving the evolution of new technologies. Nowadays, their usage as an integral part in several modern applications on a variable size scale is apparent, encompassing miniature and portable devices; such as in cell phones, laptops, medium scale; such as in hybrid (HV), plug-in hybrid (PHEV) and electric vehicles (EV) up to large scale stationary and grid applications [1,4]. As one of the scalable battery systems, lithium-ion batteries have been at the forefront in attracting great interests since the great discovery and ingenious use of Li-ion intercalation compounds as negative electrodes [1]. Although the capacities (measure of electrons number obtained from the active material) offered by most common lithium-ion intercalation compounds are lower than those provided by the Li metal (i.e., 372 mAh g^−1^, 837 mAh cm^−3^ for LiC_6_ vs 3862 mAh g^−1^, 2061 mAh cm^−3^ for Li metal), their specific energy densities were proven to be more competitive than that of other rechargeable batteries, such as nickel (Ni)–metal hydride, Ni-cadmium (Cd), and lead (Pb)–acid (about 2.5 times). They also provide higher specific power and have had long durability [1]. The fascinating advancements in Li-ion batteries have resulted in a state of the art battery which uses graphitized carbon as the anode, a transition metal oxide as the cathode, coupled such that 240 Wh kg^−1^, 640 Wh L^−1^ are provided for thousands of cycles [1]. The wide spread use of Li-ion battery, has been and remains a testament for the numerous breakthroughs and technical advancements made thus far.

One of the main challenges that current rechargeable battery technologies face is their inability to maintain energy and power densities sufficient to meet those demanded by their applications. In fact, the gap between the energy storage needs and what state of the art systems are capable of providing is increasing. This ever increasing gap has been a persistent force that drove many of the innovations made over the last 40 years [1]. For example, lithium batteries using lithium metal anodes have attracted attention as a candidate to fill up the aforementioned gap. However, this system suffers from the intrinsic property of lithium to form needle-like lithium crystals, known as dendrites, when it is plated. These grow with subsequent plating/stripping cycles, resulting in an internal short circuit and fire hazards [5–6]. While effective countermeasures are still being discussed [6], the birth of the first commercial Li-ion battery in the early 1990s was catalyzed by the need to overcome these challenges. This resulted in a decline in further technical progresses and commercialization of what was referred to as the “ultimate lithium metal anode”. If we wish to move forward towards achieving an ultimate energy density goal, technologies beyond Li-ion batteries would be needed. Fortunately, in recent years, such desire has led to an increased interest in other chemistries that employ metals poised to provide higher energy densities without compromising the safety of the battery. For example metals such as magnesium and aluminum were proposed [1,7]. Magnesium metal has been attracting an increased attention as it possesses higher volumetric capacities than lithium metal, i.e., 3832 mAh cm^−3^ vs 2061 mAh cm^−3^ for lithium. It may also provide an opportunity for battery cost reductions due to its natural abundance in the earth crust (5th most abundant element) [7–8]. More importantly, despite the fact that magnesium metal is not competitive with lithium metal on both specific capacity (2205 mAh g^−1^ vs 3862 mAh g^−1^ for lithium) and redox potential levels (−2.3 V compared to −3.0 V for Li vs NHE), the electrochemical processes related to its reversible plating/stripping have demonstrated the absence of dentrites formation which has thus far alleviated safety concerns related to employing it as a negative electrode in batteries [9]. However, several technical challenges that hamper the commercialization of rechargeable magnesium batteries are currently present. In fact, the absence of practical electrolytes and cathodes has confined demonstrations of rechargeable magnesium batteries to research laboratories. That is, low gravimetric energy densities in the order of few hundreds watt hour per kilogram and a limited shown durability coupled with very sluggish kinetics make magnesium batteries currently far from being practical. Fortunately, critical technical advancements geared towards overcoming the existing hurdles are made continuosly [7,9]. These, along with past and future dedicated research efforts, would play a vital role in enabling the maturity and readiness of rechargeable magnesium battery technologies. Herein, a technical review of rechargeable magnesium batteries is provided with focus on the most recent scientific advancements. We provide a brief summary of past breakthroughs as they were comprehensively reviewed elsewhere [7–10]. Keeping in-line with high academic quality, non-peer reviewed articles, patents and conference abstracts are not included. As the battery is a complex system employing several components, the review will individually address progresses related to the major components which are the anode, the electrolyte and the cathode. For each of these components, the existing hurdles are individually outlined and our suggestions for future research needs are provided.

## Review

### 1 Magnesium battery anodes

Since demonstrating the first rechargeable magnesium battery, magnesium metal has been viewed as an attractive battery anode due to the desirable traits outlined in the Introduction. Nonetheless, the undesirable reactivity of this metal coupled with a relatively highly reducing electrochemical environment remains a source of several challenges as explained in subsection 1.1 Aiming at overcoming these, magnesium ion insertion anodes have been recently proposed and demonstrated. These are explained in subsection 1.2.

#### 1.1 The magnesium metal anode

**Figure 1:**
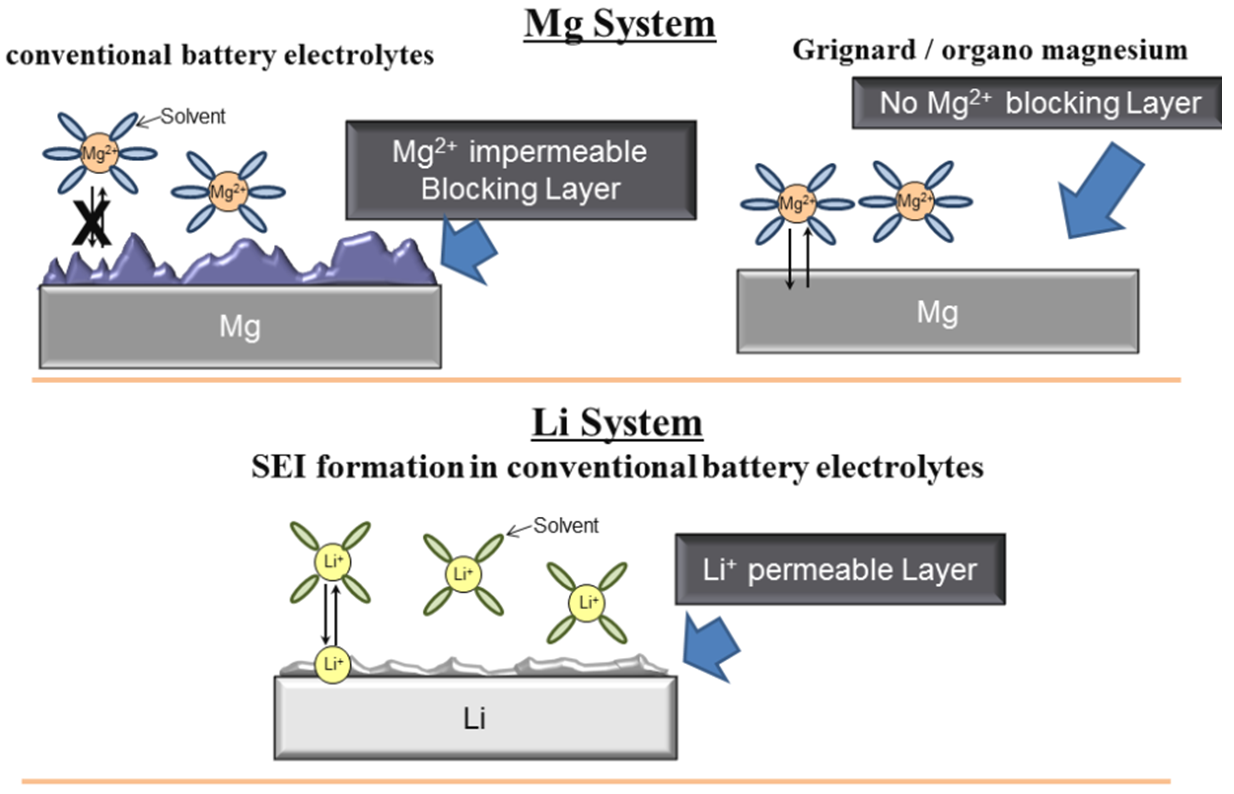
Schematic depicting a simplified image of metal–electrolyte interfaces for magnesium and lithium metals. The magnesium metal case; unlike the lithium, experiences a blocking layer formation when exposed to conventional electrolytes, i.e., ionic salts and polar solvents. No Mg passivation (bare Mg) occurs in ethereal organo-magnesium electrolytes.

When discussing the magnesium metal, the nature of its interaction with the electrolyte represents an important and complex topic. That is, interfaces formed on the metal resulting from metal–electrolyte interaction have a direct impact on electrochemical properties related to the dissolution and plating of the metal, i.e., discharge and charge of the battery. Therefore a discussion of the magnesium metal anode is primarily that of its interactions with the electrolytes. In fact, it is well established [7,9–11] that the formation of a surface layer as a result of metal–electrolyte chemical/electrochemical interaction is detrimental for reversible magnesium deposition, as it blocks the diffusion of the magnesium ions thereby preventing reversible electrochemical dissolution and plating from taking place (for illustration see Figure 1). While the nature of this “blocking” layer has not been fully established, its formation was explained by the instability of the electrolytes in proximity of the magnesium metal [11], namely electrolyte decomposition occurred. The passivating nature of this layer is astonishingly in stark contrast to what is observed when analogous electrolytes are in contact with lithium metal as the layer formed, referred to as SEI or solid electrolyte interface, allows for lithium ion diffusion and was proven critical in preventing further decomposition of the electrolyte in the highly reducing environment during lithium plating [5–6].

The challenge resulting from the electrolyte decomposition at the interface of the magnesium metal has plagued the development of electrolytes for rechargeable magnesium batteries. For example, simple ionic magnesium salts such as perchlorates and tetrafluroborates were deemed unsuitable as they formed a blocking layer on the magnesium metal [9–12]. Polar aprotic solvents such carbonates and nitriles also formed a blocking layer on the magnesium metal [9–11]. This exacerbated the challenge of electrolytes development as it limited the choices of electrolytes to a handful of organo-magnesium reagents–solvents combinations which were found to suffer from several disadvantages as described in section 2. Therefore, the discovery of new electrolytes that are compatible with rechargeable magnesium batteries and carry the promise of overcoming the existing hurdles represents an important milestone in the magnesium battery R&D. Section 2 provides a review of a variety of new promising electrolytes which we have categorized based on their type and physical state.

**Figure 2:**
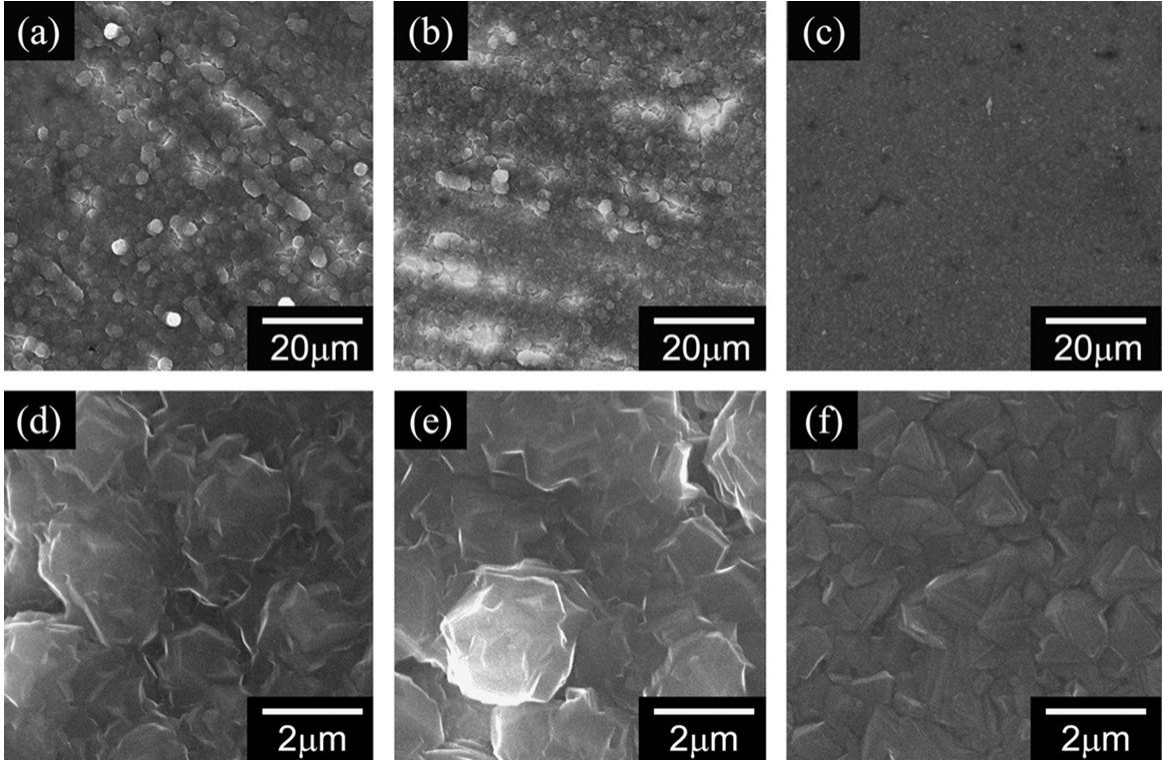
SEM images of the electrodeposited magnesium (a) 500×, 0.5 mA cm^−2^, (b) 500×, 1.0 mA cm^−2^, (c) 500×, 2.0 mA cm^−2^, (d) 5000×, 0.5 mA cm^−2^, (e) 5000×, 1.0 mA cm^−2^, and (f) 5000×, 2.0 mA cm^−2^. Reprinted with permission from [13]. Copyright 2011 Elsevier.

An important property related to the electrochemical plating of magnesium is the morphology of the magnesium deposits. Although reports related to this topic are scarce [9], they show the absence of dendritic formations following magnesium plating from organohalo-aluminate electrolytes. A recent systematic study examined the morphology of the magnesium deposits from a magnesium organohalo-aluminate complex function of deposition current densities. Although no dendritic morphologies were observed as in Figure 2, the preferred orientation of the deposits was found to depend on the current densities. For example, the deposits obtained at low current densities exhibited the (001) preferred orientation while the (100) was favored at high current densities [13]. This suggested that crystals growth of deposited magnesium is determined by the thermodynamic stability and the diffusion rates of Mg ions.

#### 1.2 Magnesium ion insertion anodes

In order to overcome limitations of the electrolytes induced by their reactivity with the magnesium metal, insertion type anodes were proposed as one potential solution. As described below, magnesium insertion anodes did offer the opportunity of using electrolytes made from magnesium ionic salts in polar aprotic solvents. However, they are currently faced with challenges caused by extremely sluggish magnesium insertion/extraction kinetics and electrode pulverization due to volume change.

The use of insertion anodes was reported by Arthur et al. [14] who sought to demonstrate the possibility of electrochemical reversible insertion/extraction of magnesium ion into Bi, Sb, Bi_0.88_Sb_0.12_ and Bi_0.55_Sb_0.45_ alloys at potentials less than 0.4 V vs Mg using an organohalo-aluminate/tetrahydrofuran electrolyte. While the highest initial specific capacity at 1 C rate was reported for the Bi_0.88_Sb_0.12_ (298 mAh g^−1^), it dropped to 215 mAh g^−1^ after 100 cycles. The smallest capacity fade with cycling was observed for the Bi anode (pulverization due to volume expansion during magnesium insertion was observed). They also provided a proof of concept for the possibility of magnesium ions insertion/extraction into Bi from magnesium bis(trifluoromethansulfonyl)imide, Mg(TFSI)_2_, in acetonitrile solvent, which are known to form a blocking layer on the magnesium metal. Reaction mechanisms of magnesium ion insertion/extraction into these anodes are currently under investigation as the interfaces likely formed on the anode surface are non- or just partially blocking.

**Figure 3:**
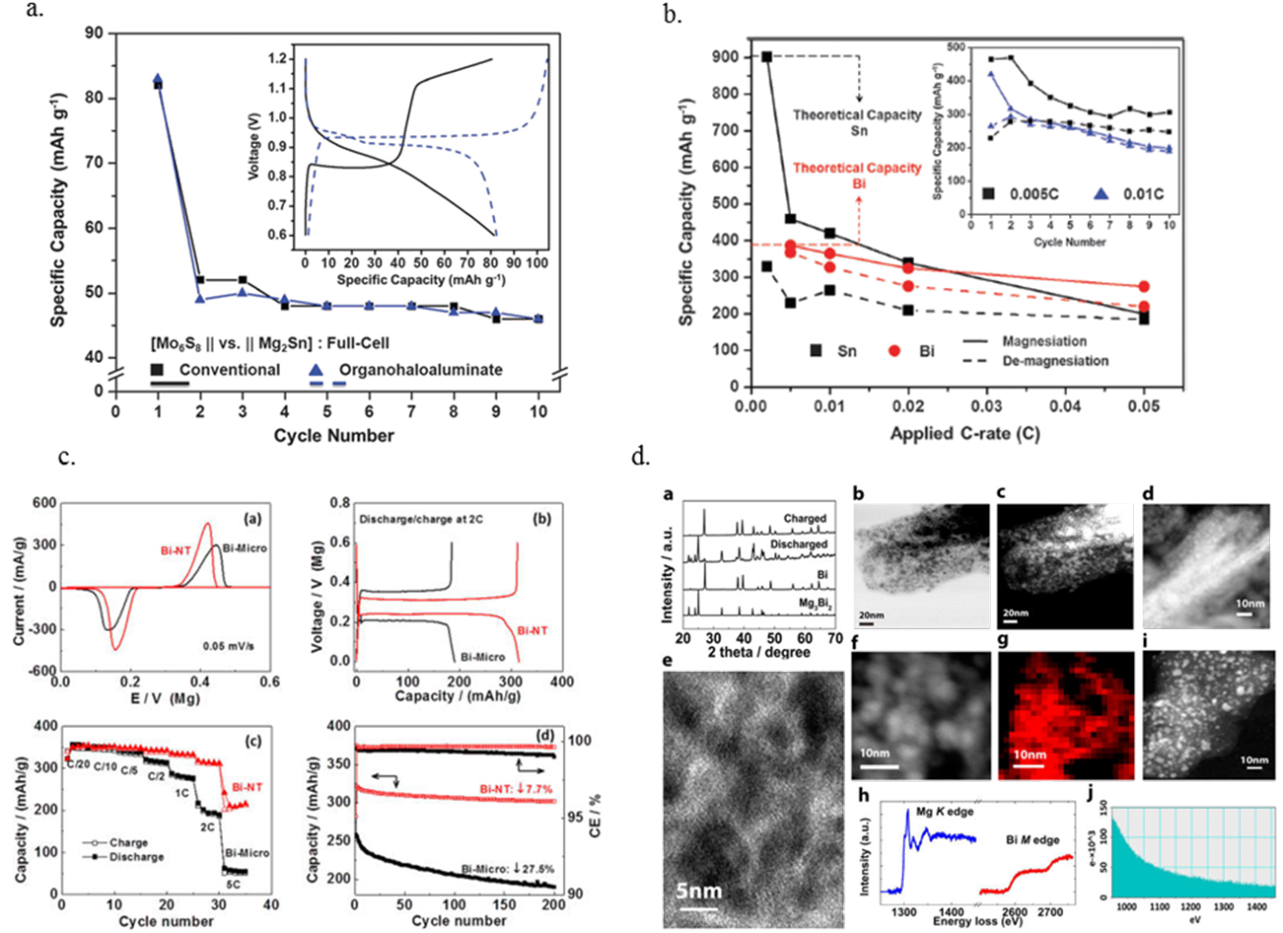
For Sn anode: a) The first 10 cycles for a Mg_2_Sn (anode), Mo_6_S_8_ (cathode) in conventional and organohalo-aluminate electrolytes, inset – 1st cycle voltage profiles; b) insertion/extraction capacities for Sn/Mg and Bi/Mg (half-cells) in an organohaloaluminate electrolyte at various C-rates. Inset – 10 cycles of a Sn/Mg half-cell at 0.005 C and 0.01 C. Figures 3a and 3b are reproduced with permission from [15]. Copyright 2013 The Royal Society of Chemistry. For Bi nanotubes anode: c) Performance comparison between Bi micro and nanotube (half cell); d) Morphology and structure evolution of Bi nanotubes during Mg insertion /extraction. Figure 3c and 3d are reprinted with permission from [16]. Copyright 2013 American Chemical Society.

Motivated by improving the capacity and lowering the insertion/extraction voltages of the magnesium ion, Singh el. al. [15] utilized Sn to demonstrate reversible and comparable anode performances in both organohalo-aluminate/tetrahydrofuran and Mg(TFSI)_2_/acetonitrile electrolytes (Figure 3a). The first insertion cycle showed a magnesiation capacity close to the theoretical value (903 mAh g^−1^ vs 384 mAh g^−1^ for Bi, ran at 0.005 C), a low working potential (0.15 V vs Mg) and lower hysteresis than that afforded by the Bi (50 mV vs 90 mV). Pulverization due to substantial volume expansion during magnesium insertion was also observed. A major challenge with these anodes is the low capacities obtained even at relatively low cycling rates. For example, the capacity when magnesium was inserted at 0.05 C rate into Bi and Sn was maintained at 70% and 20% of the theoretical values, respectively (Figure 3b).

Enhancement of magnesium ion solid state diffusion during the insertion/extraction process is expected to increase the reaction kinetics and improve the capacity retention. Shao et al. [16] recently reported a Bi anode with improved rate capabilities and capacity retention using Bi nanotubes (Figure 3c). The idea was to reciprocate the improved diffusion rates observed for Li ion insertion into nanostructured anodes; i.e., Si and Sn [17–18]. The Bi nanotubes particularly displayed improved rate capabilities, for example, when cycled at a 5 C rate, about 60% of the theoretical capacity was obtained (note that capacity retention was only shown for few cycles). Operation at 0.05 C resulted in a minimal capacity fade of 7.7% after 200 cycles. This was despite the fact that these nanotubes did not retain their structure and converted into what was described as interconnected nanoparticles upon the 1st magnesiation (Figure 3d). Interestingly, in a control experiment, the capacity retention of the nanotubes was found to be higher than that of Bi nanoparticles (fade of 16.2% after 200 cycles). Further studies examining the evolution and nature of structural and morphological transformations during magnesium ion insertion/extraction cycles would be desired.

#### 1.3 Perspectives on future developments of magnesium battery anodes

When it comes to discussing the magnesium metal, the topic is mainly about the nature of the interfaces formed. Understanding these interfacial layers, as new electrolytes are proposed, goes into the heart of enabling practical rechargeable magnesium batteries. That is, the future knowledge gained may result in discovering or even designing appropriate SEIs. This is important, as one should not forget the role the SEIs play in minimizing the decomposition of the electrolytes in Li based batteries, thereby having a direct impact on the durability of these batteries. Also, it is essential that the morphologies of the deposited magnesium, function of the electrolyte, current density and prolonged cycling continue to be examined especially as new electrolytes are emerging.

Since the great success of Li-ion batteries resulted from replacing lithium metal with the graphite anode, a similar fate may await magnesium batteries that use Mg-ion insertion anodes. What is unique about magnesium-ion insertion anodes is the possibility to reversibly insert/extract magnesium ions in conventional ionic magnesium salts, such as Mg(TFSI)_2_, dissolved in a variety of organic solvents. While the reason for this behavior has yet to be determined, one plausible explanation could be related to the thermodynamic potential of magnesium ion insertion into the host matrices. It may be possible that it occurs at higher electrochemical potentials than that of magnesium plating. Although the discovery and optimization of new materials are certainly required, several properties would need to be carefully examined in order for these anodes to become practical. First of all, it would be crucial that potential applications are considered as anodes are being developed given the very low gravimetric and volumetric capacities compared to magnesium metal. Also, the capacities of these anodes should be taken into account in the value proposition of the overall system. The second relates to the sluggish kinetics induced by the slow diffusion of magnesium ions. Indeed, the Li-ion battery literature is rich with innovative strategies proven effective in increasing the rate capabilities, some of which might be adoptable to magnesium insertion type anodes. The third point relates to examining the presence and nature of possible insertion anode–electrolyte interfaces which may form electrochemically/chemically. Not only these impact the rate of magnesium ions insertion/extraction, but also provide valuable insight into potential interfaces that may enable facile magnesium ion diffusion, that up to this point, remain unknown.

### 2 Magnesium battery electrolytes: State of the art and design guiding principles

The earliest report on a magnesium battery electrolyte that enables reversible electrochemical dissolution/plating of magnesium dates back to the 1990s. Gregory et al. [12] proposed several electrolytes for a rechargeable magnesium battery initially guided by earlier reports on successfully plating magnesium metal from the electrolysis of Grignard reagents. These included Grignard, aminomagnesium chlorides and organoborate reagents in ethereal solvents. They screened electrolytes based on the possibility of reversibly electrodepositing/stripping magnesium metal and intercalating magnesium ions into host compounds which served as cathodes. The results were used to guide the selection of the most promising electrolytes subsequently used in demonstrating the first rechargeable magnesium battery. Key findings included: 1) Ionic salts such as Mg(BF_4_)_2_ and Mg(ClO_4_)_2_ enabled reversible magnesium insertion into host materials, however formed passivating film on the magnesium metal. This observation led them to correlate the ionicity of the salt, measured by the partial charge of the magnesium ion, to its compatibility with the magnesium metal, i.e., salts with higher charge on the magnesium ion show low or no compatibility with magnesium. 2) Alkyl Grignard reagents had undesirable chemical reactivity towards the cathodes and were deemed inappropriate for battery demonstrations. 3) Some of the organoborates (magnesium dibutyldiphenyl Mg(BPh_2_Bu_2_)_2_ and tributylphenyl Mg(BPhBu_3_)_2_) supported reversible magnesium stripping/plating and Mg ions insertion into cathodes. These were also chemically inert towards the cathodes and had a high solubility in tetrahydrofuran (THF) solvent (>0.4 molar). Other organoborates were excluded from further studies due to their reactivity with the cathode (Mg(BBu_4_)_2_) or low solubility (Mg(BPh_3_Bu)_2_ ≈ 0.1 M, Mg(BPh_4_)_2_ < 0.01 M).

Mg(BPh_2_Bu_2_)_2_ was used in the first demonstration of a rechargeable magnesium battery. Unfortunately the battery was operated at less than 2 V due to the low stability of Mg(BPh_2_Bu_2_)_2_ against electrochemical oxidation. Substitution of the boron with aluminum or the hydrogen in the aromatic rings with fluoride (as was demonstrated recently [8]) was proposed to help enhance its oxidative stability. Note that a recent report by Muldoon et al. [19] confirmed the low solubility of Mg(BPh_4_)_2_, Mg(BPh_3_Bu)_2_ and found that Mg(BPh_3_Bu)_2_ had similar oxidative stability and magnesium metal compatibility as Mg(BPh_2_Bu_2_)_2_.

In the early 2000, Aurbach et al. reported a breakthrough which constituted preparing an electrolyte with higher oxidative stability (2.5 V vs Mg) than the organoborates (1.9 V vs Mg for Mg(BPh_2_Bu_2_)_2_) by combining a Grignard reagent with aluminum-based Lewis acids such as AlCl_3−_*_n_*R*_n_*; where R was an alkyl [20]. Their concept was to strengthen the Mg–C bond in the Grignard reagent, through increasing its ionic character, by adding an electron withdrawing Lewis acid. The optimized compositions of the organohalo-aluminate electrolytes enabled highly reversible magnesium deposition/stripping (100% coulombic efficiency) and insertion into host cathodes with faster insertion kinetics than the organoborates [21–22]. Their approach of using a Lewis base/Lewis acid combination to prepare magnesium battery electrolytes provided a foundation that was used to prepare other organohalo-aluminate electrolytes with high stability against electrochemical oxidation. Subsequent extensive studies by the same group reported other electrolytes based on combining Grignard reagents with other Lewis acids, such as those boron based. The electrochemical performances for the ones based on aluminum Lewis acids outperformed those boron based [22]. Later reports by Aurbach et al. demonstrated another organohalo-aluminate electrolyte that, while possessing the optimized electrochemical performance of those reported previously, had an impressive stability against oxidation exceeding 3.0 V vs Mg. The idea was to remove the source of β-H elimination, believed to be causing the lower oxidative stability in previous electrolytes, by exchanging the Grignard alkyl ligand with a phenyl group [23]. More recently, other organohalo-aluminate electrolytes with high oxidative stability were reported by other groups. Examples included adding AlCl_3_ to less nucleophilic amidomagnesium chloride (hexamethyldisilazide) [24]; previously known to allow for reversible magnesium deposition/stripping [25]. Kim et al. [24] found that the crystalized product outperformed the in situ produced electrolyte (oxidative stability of up to 3.2 V vs Mg and higher magnesium deposition/stripping current densities). Another approach used a phenylmagnesium chloride combination with a boron-based Lewis acid in tetrahydrofuran such as tris(pentafluorophenyl)borane [8] or (tri(3,5-dimethylphenyl borane) [26] to form stable electrolytes up to 3.7 V and 3.5 V, respectively. Unfortunately, all these electrolytes, while demonstrated with impressive electrochemical stability windows, reversible magnesium dissolution/deposition properties, and high bulk conductivity (i.e., 2 mS cm^−1^), share several critical draw backs which are: 1) The presence of chloride; which is an integral part in the make of these salts/complexes. This was found to cause severe corrosion of non-noble metals that becomes apparent at potentials exceeding 2 V vs Mg [7–8,26]. This is problematic as it prohibits using materials such as steel or aluminum as current collectors when using these electrolytes. 2) Tetrahydrofuran is the preferred solvent which is undesirable due to its high volatility and tendency to form peroxides. Aurbach et al. [22] demonstrated optimized compositions obtained from mixing the electrolytes they developed with less volatile ethers such as tetraglymes. However, tetrahydrofuran was still part of the best performing electrolytes; albeit in lesser amounts. 3) Although no systematic studies addressing the extent of the electrolytes’ air sensitivity exist, it is likely that they would degrade following exposure to air.

Motivated by overcoming the above problems, research efforts recently started shifting from typical organohalo-aluminates/organoborate-based electrolytes, and the discovery of new systems belonging to a variety of different reagents became of interest. In the next subsections, we review and present these new electrolytes based on their type and physical state. Table 1 summarizes the properties of representative electrolytes classified based on their types. Only those that enable highly reversible magnesium deposition and stripping (i.e., >80% coulombic efficiency) are shown.

**Table 1:** Summary of Mg battery electrolytes based on their types. Properties of representative examples, including reported stability against oxidation *E*_ox_, are provided.

electrolyte type	composition	*E*_ox_ on Pt vs Mg^2+^/Mg	solvent	remarks	ref.

Liquid State					

organo/ organo-halo					

organo borates	Mg(BPh_2_Bu_2_)_2_	1.9	THF		[12,19]
	2:1 PhMgCl: Me_3_B	3.5	THF	*E*_ox_ = 2.2 V on SS	[26]
	(Mg_2_(µ-Cl)_3_·6THF)[B(C_6_F_5_)_3_Ph] Ph = phenyl, Bu = butyl, Me = methyl	3.7	THF	*E*_ox_ = 2.2 V on SS	[8]
Grignard halo-aluminate	Mg(AlCl_3-_*_n_*R*_n_*R')_2_ R, R' = alkyl or aryl	2.2	THF/glymes	optimum in THF	[20–22]
	2:1 RMgCl:AlCl_3_ R = phenyl	3.2	THF/glymes	*E*_ox_ = 2.2 V on SS	[23]

inorganic ionic salts non-halide based					

borohydrides	1:X Mg(BH_4_)_2_:LiBH_4_, X = 0–6	1.7	monoglyme/ diglyme	non-corrosive *E*_ox_ = 2.2 on SS	[27–28]

non-Grignard halo-aluminates					

phenolates & aloxides	2:1 ROMgCl:AlCl_3_			less air sensitive	
	R = phenyl alkyl	2.6	THF		[29]
	R = phenyl fluoroalkyl	2.9	THF		[30]
	6:1 ROMgCl:AlCl_3_ R = Me_3_SiO	2.5	THF		[31]
amido based	3:1 (HMDS)MgCl:AlCl_3_ 1:2 Mg(HMDS)_2_:AlCl_3_ HMDS = hexamethyldisilazide	3.2 3.5	THF diglyme	*E*_ox_ = 2.2 V on SS *E*_ox_ = 2.6 V on SS low nucleophilicity	[24] [32]
inorganic halide based	2:1 MgCl_2_:AlCl_3_	3.1, 3.4	monoglyme		[33–34]
	2:1 MgCl_2_:AlCl_4-_*_n_*R*_n_* R = alkyl,aryl	2.9	THF	*E*_ox_ = 1.8 V on SS low nucleophilicity	[34]

icosahedral boron cluster					

carboranyl Mg salt	1-(1,7-C_2_B_10_H_11_) MgCl	3.3	THF	least corrosive *E*_ox_ > 3.0 V on SS, Al	[35]

Solid State					

gel polymer	Mg(AlCl_2_EtBu)_2_	2.5	tetraglyme/ PVDF	3.7 mS cm^−1^ at 25 °C	[36]
inorganic salts	Mg(BH_4_)(NH_2_)	>3.0	none	10^−3^ mS cm^−1^ 150 °C coulombic efficiency <50%	[37]

#### 2.1 Liquid electrolytes

Given the reactivity of magnesium metal towards most solvents such as carbonates, sulfoxides and nitriles, ethers have been the solvents of choice. New liquid electrolytes are reviewed below with emphasis on those that are tetrahydrofuran-free. We also summarize recent information reported on the nature of the electroactive species in typical organohalo-aluminates and in some of the new electrolytes.

**2.1.1 Inorganic ionic salts:** Until very recently, it has been generally accepted that simple ionic salts such as Mg(TFSI)_2_ and Mg(ClO_4_)_2_ are incompatible with magnesium metal (see the Introduction section). Motivated by solving the corrosion problem caused by chloride ions and eliminating tetrahydrofuran as a solvent/cosolvent, Mohtadi et al. [27] proposed a magnesium borohydride based electrolyte for the magnesium battery. The premise of their concept was that the BH_4_^−^ ion, being a relatively strong reducing agent, could withstand the reducing environment of the magnesium anode. Their results demonstrated the first inorganic, halide free, and relatively ionic salt that could reversibly deposit and strip magnesium using magnesium borohydride. Indeed, the work confirmed that ionic salts could be made compatible with the magnesium metal if the anion in the salt has sufficient reductive stability (note that this was also the first time to show Mg plating possibility in a BH_4_^−^-containing system, as an old report on Mg plating using electrolysis (on Cu cathode and Al anode) of a MgBr_2_, LiBH_4_ mixture in diethylether/tetrahydrofuran showed lots of boron impurities, likely generated from the electrolysis side reaction. No information supporting Mg(BH_4_)_2_ formation were given [38]).

**Figure 4:**
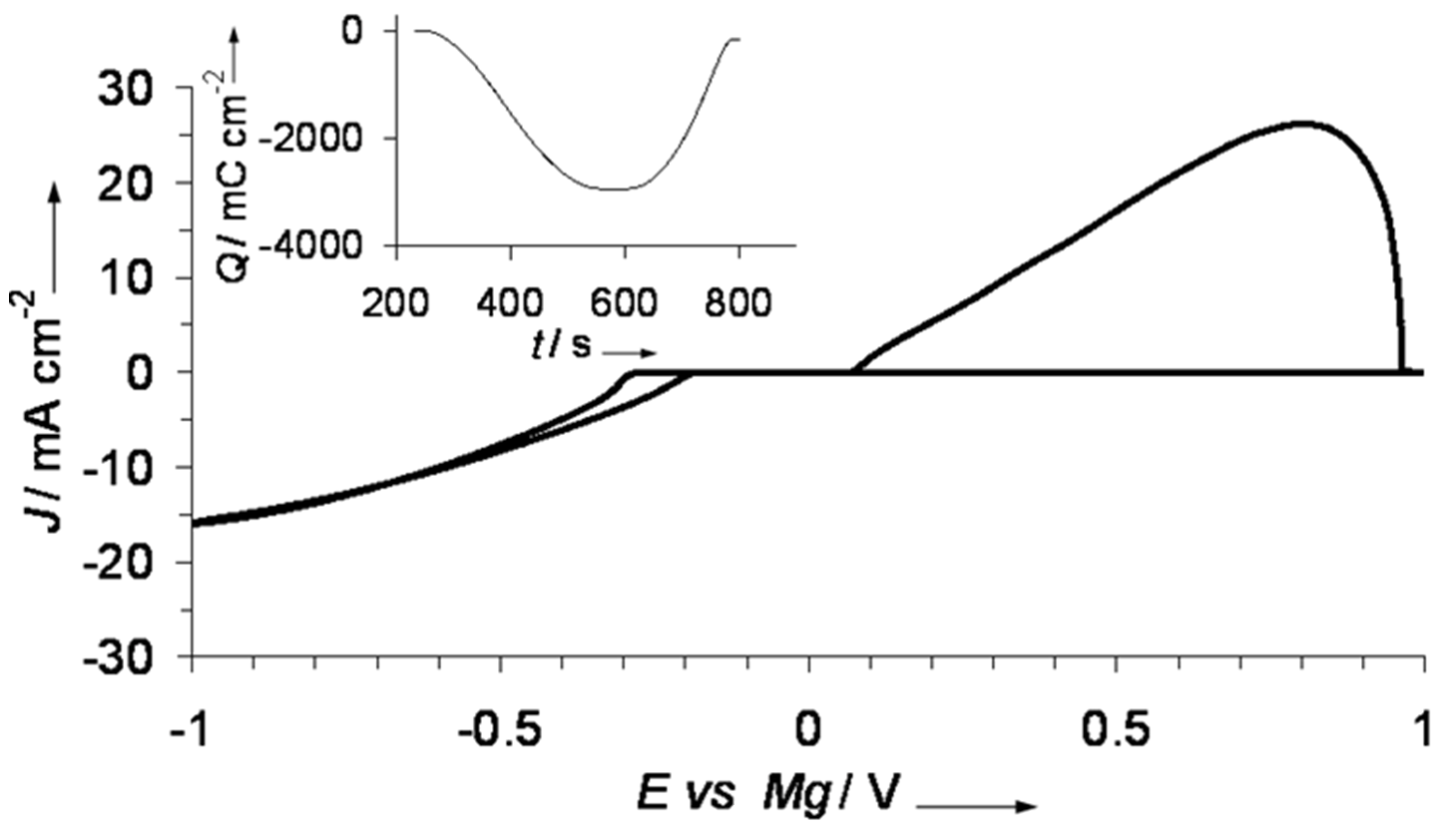
Cyclic voltammogram for LiBH_4_ (0.6 M)/Mg(BH_4_)_2_ (0.18 M) in DME, (inset shows deposition/stripping charge balance). Reprinted from [27] with permission. Copyright © 2012, Wiley-VCH Verlag GmbH & Co. KGaA, Weinheim.

Mohtadi et al. [27] also developed a magnesium borohydride–lithium borohydride electrolyte in dimethoxyethane (DME) solvent with a reversible magnesium deposition/stripping at high coulombic efficiency (94%), high current densities (25 mA cm^−2^ stripping peak current) and low deposition overpotentials (−0.3 V) as shown in Figure 4. The stability against electrochemical oxidation was 1.7, 2.2 and 2.3 V (vs Mg) on platinum, stainless steel and glassy carbon electrodes, respectively. As the borohydride electrolytes are not corrosive, these stability trends are opposite of those observed for other magnesium electrolytes. The higher stability of the borohydride on a non-noble metal suggests catalytic effects of platinum on BH_4_^−^ decomposition. Until this point, the borohydride electrolytes remain the only ionic and halide free salts that are highly compatible with magnesium metal.

**2.1.2 Non-Grignard-based haloaluminate reagents:** In order to increase the stability of the electrolytes in air, avoiding the use of Grignard reagents is needed (i.e., RMgCl or R_2_Mg Lewis base). Wang et al. [29] used phenolates to prepare new electrolytes (ROMgCl) with improved air stability, i.e., due to the stronger bond in Mg–O compared to Mg–C. Three phenolate electrolytes exhibiting good Mg reversibility were prepared, however the conductivity and electrochemical oxidative stability were dependent on the alkyl group. The highest conductivity and oxidative stability measured on a platinum electrode were observed for a 0.5 M 2:1 2-*tert*-butyl-4-methylphenolate magnesium chloride:AlCl_3_ in tetrahydrofuran at 2.56 mS cm^−1^ and 2.6 V vs Mg, respectively. Reversible magnesium deposition/stripping, albeit with an increased overpotential, was observed for the same electrolyte following exposure to air for three hours. A new systematic study by Nelson et al. [30] examined the oxidative stability of phenolates as a function of the substituents on the phenyl ring. Several electrolytes were prepared with electron withdrawing (pentafluoro, trifluoromethyl) or donating (methoxy) substituents. Oxidative stability, measured on a platinum electrode, of up to 2.9 V vs Mg was obtained for a 2:1 4-(trifluoromethyl)-phenolate magnesium:AlCl_3_ in tetrahydrofuran. This electrolyte supported reversible magnesium deposition/stripping and had a high conductivity (2.44 mS cm^−1^). However, some degradation in the electrochemical performance was observed following exposure to air for six hours (i.e., lower current densities and higher overpotentials). Unfortunately, this suggested the instability of the phenolates upon prolonged exposure to air. Electrolytes prepared by the replacement of the phenolates with alkoxides were reported by Liao et al. [31], who prepared three new butoxy and siloxy based electrolytes. Their interest was to access the vast numbers of ligands offered by the alkoxides such that electrolytes with improved oxidative stability could be prepared. In the absence of AlCl_3_ Lewis acid, the alkoxides had higher solubility in tetrahydrofuran than the phenolates and supported reversible Mg deposition/stripping. However, the addition of AlCl_3_ was necessary to improve their oxidative stability (one sixth an equivalent AlCl_3_ was added to mitigate its negative impact on the solubility of the alkoxides). For example, the addition of AlCl_3_ increased the oxidative stability of Me_3_SiO–MgCl from 1.95 to 2.5 V vs Mg (on a platinum electrode). Both phenolate- and alkoxide-based electrolytes supported reversible magnesium ion insertion in the Chevrel phase Mo_6_S_8_ cathode [29–31].

**Figure 5:**
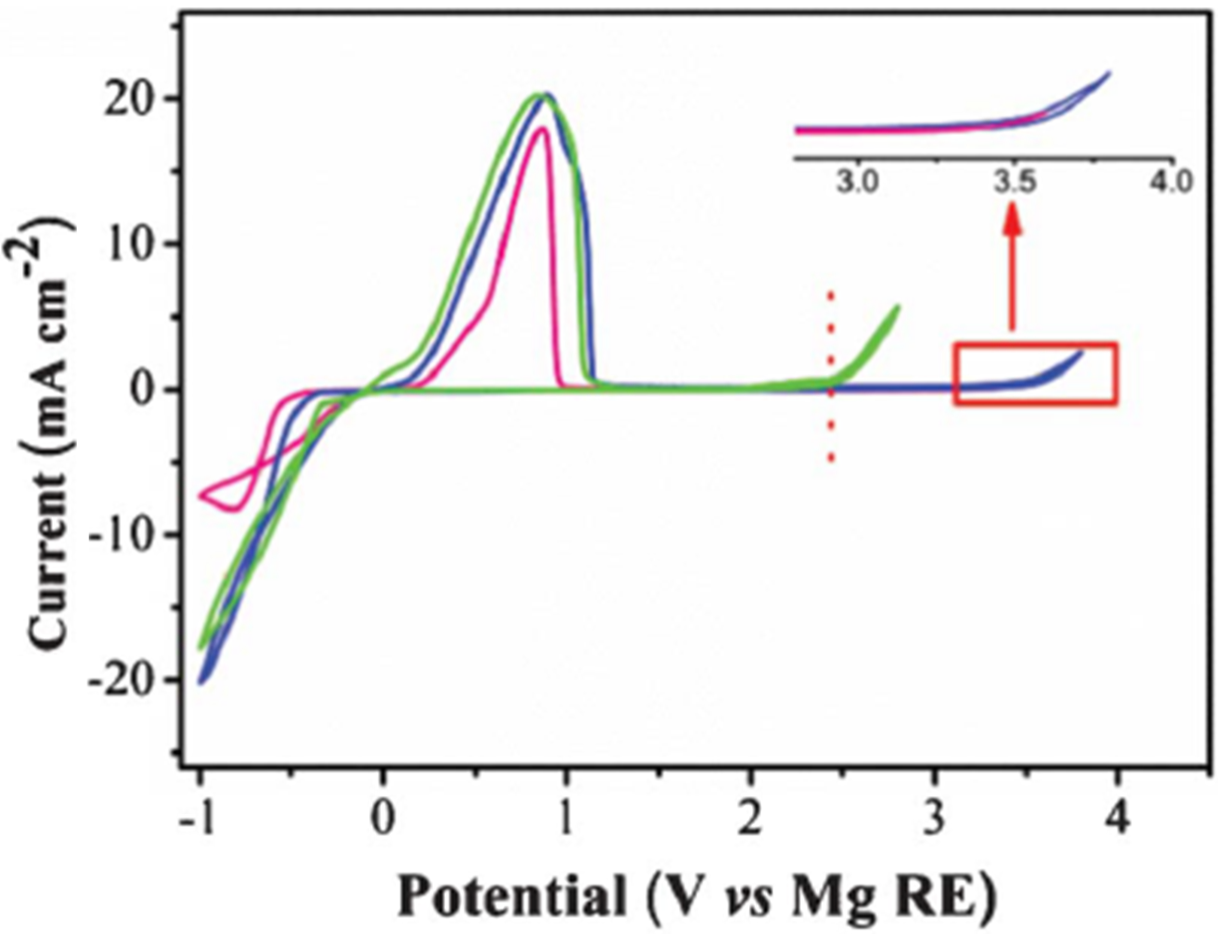
Cyclic voltammograms of the Mg deposition/dissolution in 0.25 M THF solution containing as-prepared (HMDS)_2_Mg–2AlCl_3_ (blue) and redissolved crystals [Mg_2_(µ-Cl)_3·_6THF][HMDSAlCl_3_]·THF (pink). The as-prepared [(iPr_2_N)_2_Mg–2AlCl_3_] is also shown (green). Reproduced with permission from [32]. Copyright 2013 The Royal Society of Chemistry.

As mentioned in the introduction, Kim et al. [24] reported a less nucleophilic 3:1 (hexamethyldisilazide)MgCl:AlCl_3_ electrolyte where the crystallized product had an oxidative stability of 3.2 V vs Mg on a platinum electrode (note that crystallization was necessary to achieve this performance). More recently, Zhao-Krager et. al [32], also motivated by the lower nucleophilicity of sterically hindered amides, used magnesium bisamides to prepare two electrolytes by reacting magnesium bis(diisopropyl)amide (iPr_2_N) and magnesium bis(hexamethyldisilazide) (HMDS) with two equivalents of AlCl_3_. As shown in Figure 5, the HMDS based electrolyte (both as prepared and crystallized) exhibited the best electrochemical performance and had a higher oxidative stability (3.3 V vs Mg) than the iPr_2_N based. Interestingly, the structure of the crystallized material obtained from the Mg(HMDS)_2_:2AlCl_3_ was the same as that reported by Kim et al. [24] for the (HMDS)MgCl:AlCl_3_. Another recent progress on using non-Grignard halo-aluminate electrolytes was reported by Doe et al. [33], who showed the possibility of magnesium deposition/stripping at high coulombic efficiencies simply from MgCl_2_, AlCl_3_ mixture in tetrahydrofuran. Similar results were concurrently reported by Liu et al., who also showed the mixture to have a very low nucleophilicity [34]. Unfortunately, the MgCl_2_ electrolytes were found to be very corrosive; i.e., stability on stainless steel was as low as 1.8 V vs Mg [34]. What is notable about the Mg(HMDS)_2_:AlCl_3_ and MgCl_2_:AlCl_3_ systems is that the in situ products exhibited wide electrochemical windows and high electrochemical performances thereby eliminating the necessity of additional crystallization steps.

**Figure 6:**
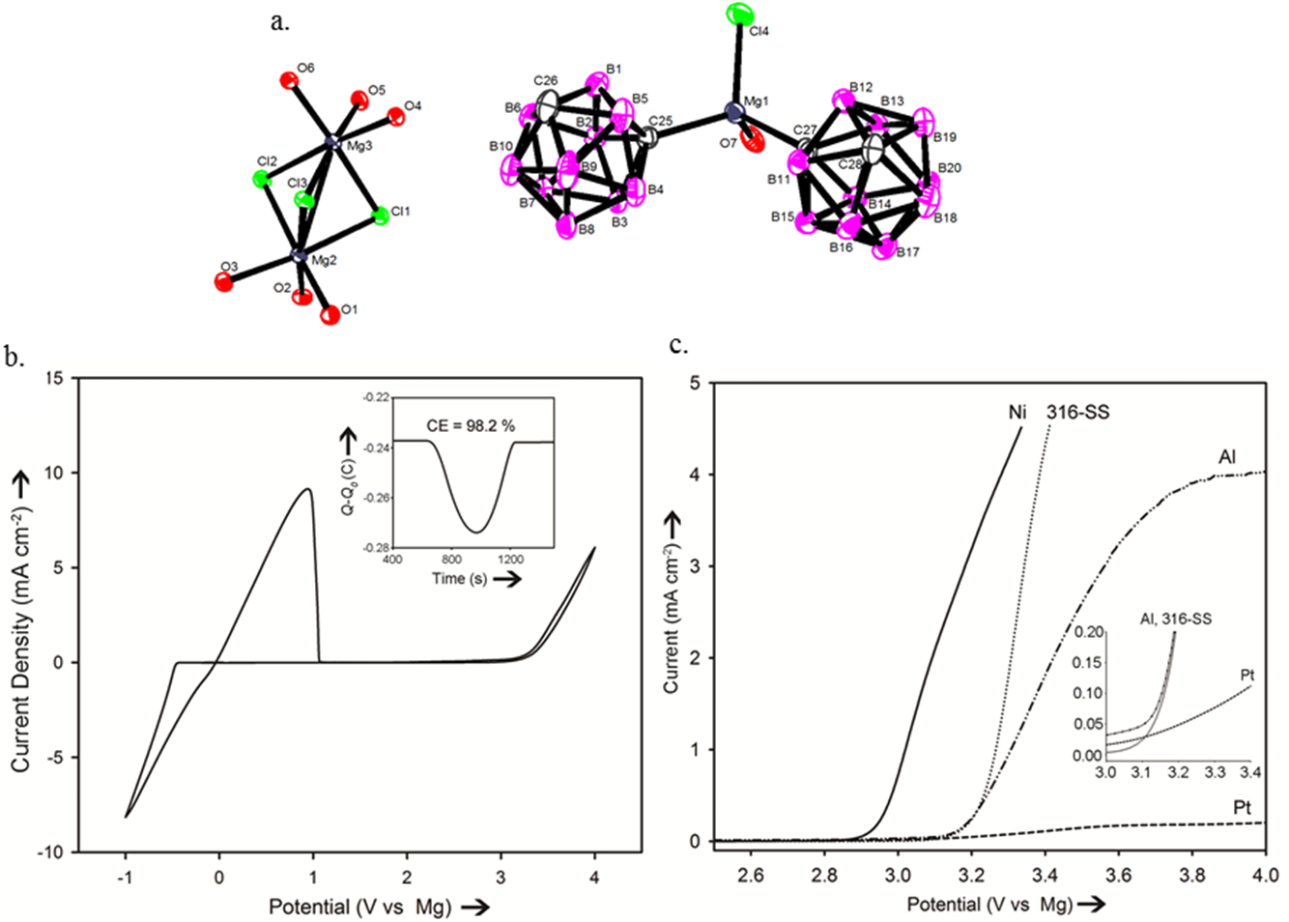
a) X-ray crystal structure of 1-(1,7-C_2_B_10_H_11_) MgCl. Hydrogen atoms and THF carbon atoms are omitted for clarity. b) Cyclic voltammogram in THF solution (inset: charge balance of Mg deposition/stripping). c) Linear sweep voltammograms of Mg(C_2_B_10_H_11_)Cl/THF on Pt, Stainless steel (316-SS), Ni and Al electrodes (inset: expanded view of the oxidation onset). Reprinted with permission from [35]. Copyright 2012 Wiley-VCH Verlag GmbH & Co. KGaA, Weinheim.

**2.1.3 New design strategies for forming high stability electrolytes:** As was described before, the high electrochemical oxidative stability of magnesium electrolytes has been primarily enabled by the formation of strong Al–C, Al–N or B–C bonds (formed by the addition of appropriate Lewis acids). A very recent study by Carter et al. [35] targeted to increase the oxidative stability of the Mg(BH_4_)_2_ electrolytes by strengthening the B–H bond through forming 3-dimensional B–B bonds as in icosahedral boron clusters (*closo*-boranes). As such, the group exploited the high oxidative and thermal stability of *closo*-boranes to prepare electrolytes with wide electrochemical stability window. The results demonstrated a novel carboranyl magnesium chloride electrolyte (1-(1,7-C_2_B_10_H_11_) MgCl) that is compatible with magnesium metal, possesses high oxidative stability (3.3 V vs Mg), and to date, exhibits the lowest tendency to corrode non-noble metals observed from a chloride bearing electrolyte (Figure 6). What was also notable is that the stable anion consisted of a magnesium Mg–C center as shown in Figure 6 below, indicating unique effects of the carborane scaffold. The cation was found to be the Mg_2_Cl_3_^+^ observed before for other systems (see section 2.1.5). This was the first time to show that electrolytes with a wide electrochemical window could be prepared beyond known approaches that use Lewis base, Lewis acid systems. This work opens new horizons for designing highly stable magnesium battery electrolytes.

**2.1.4 Tetrahydrofuran-free electrolytes:** Given the volatile nature of tetrahydrofuran (143 mm Hg at 20 °C, and 66 °C boiling temperature), it would be vital to discover electrolytes that are tetrahydrofuran-free. Aurbach et al. [22] demonstrated that they could utilize their organohalo-aluminate electrolytes in solvent mixtures of tetrahydrofuran and longer chain ethers such as tetragylmes without inducing losses in their electrochemical performances. However, it would be hard to fully eliminate the presence of tetrahydrofuran as the organohalo-aluminates based on Grignard reagents tended to have a favorable performance in this solvent. Therefore, an important step in the development of the electrolytes would be demonstrating optimum performances in a tetrahydrofuran-free system. This may be enabled using electrolytes beyond those that use a Grignard Lewis base/Lewis acid reaction. In fact, highly reversible performance from a magnesium borohydride, lithium borohydride electrolyte, developed by Mohtadi et al. [27], was found in dimethoxyethane (monoglyme) solution. Actually, the magnesium borohydride had far superior electrochemical performance in monoglyme than that observed in tetrahydrofuran. Very recently, highly reversible performance (100% coulombic efficiency) for a similar borohydride electrolyte was demonstrated in diglyme solvent [28]. High cycling magnesium deposition/stripping efficiencies approaching 100% were reported for 0.35 M (HMDS)_2_Mg–2AlCl_3_ in diglyme solution where high oxidative stability above 3.5 V vs Mg was obtained. Interestingly, the electrolyte stability measured on a stainless steel electrode was 0.4 V higher than that of a similar system in tetrahydrofuran (2.2 V vs Mg) [8]. At this time, all the electrolytes use ethereal solvents which are more or less volatile. An attractive choice for eliminating the safety hazards of ethers would be using ionic liquids due to their very low volatility. Reversible magnesium deposition/ dissolution from phenyl magnesium bromide [39] and alkylmagnesium bromide [40] was shown in ionic liquid solvents. The caveat was that tetrahydrofuran was used as a cosolvent and as discussed earlier, a shift from Grignard reagents is hence necessary to allow for more flexibility in the solvent selection. Nuli et al. [41] reported reversible magnesium plating using conventional salts such magnesium triflate (Mg(CF_3_SO_3_)_2_) in imidazolium-based ionic liquids. However, magnesium metal passivation was reported to take place [39,42].

**2.1.5 On the electroactive species:** In the case of typical organohalo-aluminate electrolytes, formed following the reaction between a Grignard reagent and AlCl_3_, it has been generally accepted that the magnesium charge carriers in the bulk are magnesium-chloride bonded ions existing as monomeric (MgCl^+^) and/or dimeric (Mg_2_Cl_3_^+^) species [43]. Kim et al. showed that Mg_2_Cl_3_^+^ is one of the electroactive species present in 3:1 (HMDS)MgCl:AlCl_3_ [24]. Studies on organoborates (crystallized out of their synthesis solution) suggested similar electroactive species as those in the organohalo-aluminates, i.e., MgR^+^ and Mg(BR_4_)^+^, R = alkyl or aryl [44]. Given that the organohalo-aluminate electrolytes are by far the most established, detailed studies exist which were concerned with identifying the nature of magnesium species, in both the bulk and at the interface of magnesium metal–electrolyte. As the organohalo-aluminates were reviewed extensively [10], the discussion here is focused on the most recent studies concerning these. The discovery of new types of magnesium ion electroactive species, which enable reversible magnesium plating, is important for advancing the research and development of magnesium battery electrolytes. Below, we shed light on the nature of the different species suggested for the new electrolytes per the available information.

**a. Grignard organohalo-aluminate systems:** The nature of the electroactive species present at equilibrium in the bulk solution and at the magnesium metal–electrolyte interface during magnesium plating were studied previously [43–44]. For the Mg(AlCl_4−_*_n_*R*_n_*)_2_ electrolyte, the presence of the adsorbed intermediate MgCl^+^·5THF at the metal surface during the deposition of magnesium was suggested. More recently, the presence of an intermediate during magnesium deposition from a 1:2 molar RMgCl:R_2_AlCl/THF; R = C_2_H_5_, was observed by Arthur [45] and Benmayza et al. [46] using the magnesium K-edge in an in operando soft X-ray spectroscopy. Their results, combined with the transport properties of the magnesium species, also suggested the interfacial electroactive species to be MgCl^+^·5THF. The dimeric Mg_2_Cl_3_^+^ species present in the bulk, was discounted from being electrochemically active at the interface during magnesium deposition. Another important result was related to the measured low transport numbers of the magnesium ions. For example, the diffusion coefficient of the magnesium ionic species (i.e., Mg_2_Cl_3_^+^) was very low (2.26 × 10^−7^ cm^2^ s^−1^ in 0.2 M solution which is 10 times lower than that observed in 1 M LiPF_6_-based electrolyte). Interestingly, the transference number *t*^+^, which determines the rate at which reversible magnesium deposition/stripping takes place, ranged between 0.018–0.19 at 0.40–0.15 M, respectively. This astonishing reduction in *t*^+^ values with increasing the electrolyte concentration was attributed to lowered mobility of the dimeric magnesium ions and an increased number of counter and non-magnesium ions at high Lewis acid concentrations. This study helped to provide a better understanding of the electrochemical and transport properties in this complex system.

**b. Non-Grignard-based electrolyte systems:** Indeed, the magnesium borohydride electrolytes offered new electroactive species beyond known monomeric, dimeric Mg–Cl and RMg^+^ species, present in the organohalo-aluminate and probably in the organoborate electrolytes. Guided by spectroscopic analyses of the borohydride electrolytes, Mohtadi et al. [27] proposed the magnesium electroactive species to be a magnesium ion bridge bonded to a one BH_4_^−^, although the presence of magnesium ions that are solely coordinated to the solvent molecule was not discounted. The electrochemical performance was suggested to be governed by the extent of salt dissociation. The substantial improvements in the electrochemical performance as the dendicity of the solvent was increased, and following the addition of LiBH_4_, used as a source of a Lewis acid cation, further supported this hypothesis.

In the case of the amidomagnesium (HMDS) based electrolyte [32], the dimeric Mg_2_Cl_3_^+^ electroactive species was similar to that found in other Grignard- and non-Grignard-based halo-aluminate systems [24,26]. A similar species was reported for the carboranyl MgCl electrolyte [35]. Note that Mg_2_Cl_3_^+^ was also present in the crystallized products from MgCl_2_ mixtures with aluminum based Lewis acids [34].

The electroactive species was investigated for the alkoxide 6:1 *n*-butyl-OMgCl:AlCl_3_/THF electrolyte, however the crystallized product yielded an inactive species [31]. In the phenolate electrolytes, the crystallized product from the trifluoromethyl phenolate:AlCl_3_ solution contained the Mg_2_Cl_3_^+^ cation [30].

Based on the current progresses, we could summarize that two distinct species that enable reversible magnesium deposition/stripping are known: 1) The Mg_2_Cl_3_^+^ and/or MgCl^+^ in organo/non-organo haloaluminates, organoborates, and in the carboranyl electrolyte and 2) the MgBH_4_^+^ in the borohydride electrolytes. For any of the electrolytes reported thus far, there is no evidence that support Mg^2+^ presence.

As described above, for future material design of magnesium battery electrolytes, it is of significant importance to discern the electroactive species in both the bulk and at the interface between the anode and electrolyte. This is expected to be more beneficial than solely relying on optimizing the compositions/ratios of the reagents.

#### 2.2 Solid magnesium electrolytes

As explained above, the solvents known to support optimum reversible Mg deposition/stripping are volatile as they are ether-based. To overcome this challenge, one strategy would be trapping the solvent, used to solvate the magnesium ions, within a polymeric matrix. The electrolyte formed in this case is referred to as a gel electrolyte. This concept was previously applied to Li-ion battery electrolytes [5] and was adopted later for rechargeable Mg batteries [10]. Nonetheless, demonstrating a viable gel electrolyte for rechargeable magnesium batteries is not trivial as it requires using magnesium reagents/salts that enable reversible magnesium deposition/stripping, while being chemically inert towards the polymeric matrix selected. The electrolyte would also need to have an acceptable conductivity of the magnesium ions at room temperature. Another strategy, which is far more challenging, is to create a solvent free solid state medium that enables magnesium ion conduction, under practical conditions, through magnesium ion diffusion, i.e., solid state magnesium salts. While few reports exist on the formation of gel electrolytes for magnesium batteries, reports on magnesium ion conduction in the solid state media are scarce. In fact, until recently, magnesium ion conduction at values in the order of 10^−3^ mS cm^−1^ occurred only at temperatures exceeding 500 °C. A review of the developments related to both strategies, with focus on those that demonstrated viable electrolytes is presented below.

**2.2.1 Organic solid/semi solid electrolytes:** The immobilization of magnesium electrolyte in polymeric matrices such as poly(vinylene difluoride) PVDF and poly(ethylene oxide) PEO, was reported by Chusid et al. [36]. The group impregnated magnesium organohalo-aluminate salts, such as Mg(AlCl_2_EtBu)_2_ dissolved in tetrahydrofuran and tetraglyme, in both PEO and PVDF matrices. These complex solutions were found to be inert towards the polymers used and reversible magnesium deposition/stripping from these gel electrolytes was shown. The best electrochemical performance reported was for the Mg(AlCl_2_EtBu)_2_/tetraglyme/PVDF gel as high specific conductivity (3.7 mS cm^−1^ at 25 °C) was measured. This study not only showed the possibility to prepare gel electrolytes that are compatible with magnesium metal but also allowed for reversible Mg intercalation into the Chevrel phase Mo_6_S_8_ cathode. Other gel polymer electrolytes were reported [47–49]. Examples include those incorporating dispersed inorganic oxides such as nano fumed silica. The oxides were added to improve the mechanical and electrochemical properties (1 mS cm^−1^ reported at room temperature) [49]. Unfortunately, all these gel electrolytes used magnesium salts known to be incompatible with the magnesium metal. A very recent study proposed using coordinatively unsaturated metal-organic frameworks (MOFs) as nano media to immobilize magnesium phenolate and/or Mg(TFSI)_2_/triglyme electrolytes (phenolates were found to be more soluble in triglyme than in tetrahydrofuran) [50]. As the phenolates were strongly interacting with the MOF’s crystallites, addition of Mg(TFSI)_2_ (i.e., weakly coordinating anion) was necessary to achieve a good conductivity (0.25 vs 0.0006 mS cm^−1^ in just phenolates/MOF). No results addressing the compatibility with magnesium metal or oxidative stability were provided. It may be possible that this system is incompatible with the magnesium metal due to the passivating nature of Mg(TFSI)_2_.

**Figure 7:**
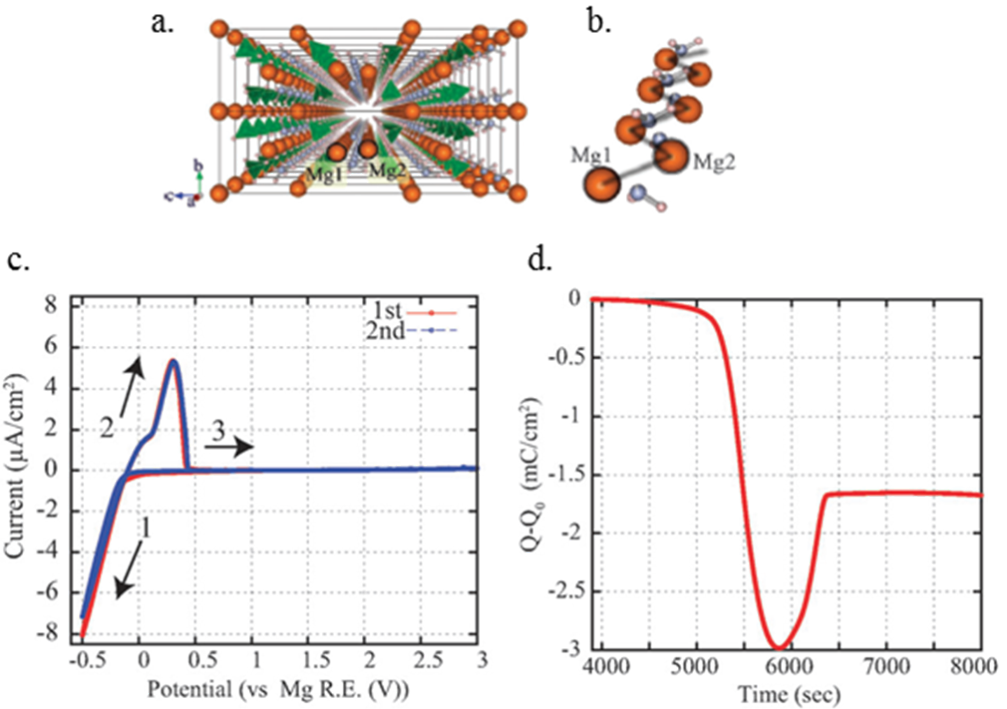
a) Crystal structure of Mg(BH_4_)(NH_2_). Atomic sizes are depicted by sphere radii. b) Mg zigzag structure from the crystal structure a). c) Cyclic voltammograms of Mg/Mg(BH_4_)(NH_2_)/Pt and, d) deposition/stripping charge balance (first cycle). Reproduced with permission from [37]. Copyright 2012 The Royal Society of Chemistry.

**2.2.2 Inorganic solid state magnesium ion conductor:** Until very recently, the observation of magnesium ion conduction in inorganic salts occurred only at temperatures exceeding 500 °C [51–52]. Recently, Matsuo et al. [53] studied the possibility of magnesium ion conduction in the high temperature phase of magnesium borohydride using first-principles molecular dynamics simulations FPMD [53–54]. The magnesium ions, present in the center of a tetrahedral cage surrounded by the BH_4_^−^ anions, were found to have limited mobility. This was attributed to the strong coulombic interactions with BH_4_^−^ resulting from the small cage size. They proposed that increasing the cage size, by partial substitution of BH_4_^−^ with the larger AlH_4_^−^, may enable magnesium ion migration, however, this was not experimentally demonstrated. Another study of borohydride-based solid state electrolytes was reported by Higashi et al. [37]. Guided by their first-principles calculations based on density functional theory (DFT), they experimentally investigated the conduction of magnesium ions in both Mg(BH_4_)_2_ and Mg(BH_4_)(NH_2_). The selection of these compounds was motivated by the ionic bonding nature of the magnesium ions, judged from the calculated Bader charge on the magnesium, and presence of cavities large enough to enable magnesium ion conduction through the hopping mechanism. They measured a conductivity of about 10^−3^ mS cm^−1^ at 150 °C for Mg(BH_4_)(NH_2_), which is three orders of magnitude higher than that of Mg(BH_4_)_2_, presumably due to the shorter distance between the two nearest Mg atoms (3.59 Å in Mg(BH_4_)(NH_2_) vs 4.32 Å in Mg(BH_4_)_2_). In addition, reversible magnesium deposition/stripping was demonstrated for the Mg(BH_4_)(NH_2_) electrolyte as shown in Figure 7. Interestingly, the oxidative stability of the Mg(BH_4_)(NH_2_) salt was found to be in excess of 3 V vs Mg at 150 °C, which is higher than that reported for liquid Mg(BH_4_)_2_–ether systems at room temperature [27]. The high ionic conductivity in Mg(BH_4_)(NH_2_), albeit at 150 °C, reversible Mg deposition/stripping and high voltage stability provide opportunities for developing practical Mg solid state electrolytes based on novel borohydride salts.

#### 2.3 Perspectives on future developments of magnesium battery electrolytes

Unlike in the the case of rechargeable lithium and sodium batteries, the development of electrolytes for rechargeable magnesium batteries has been faced with a distinct and an unavoidable challenge. This is thanks to the formation of a passivation layer upon magnesium metal exposure to numerous salts/solvents. Generally speaking, the battery system imposes several stringent requirements on the electrolytes as they represent the bridge linking the anode with the cathode. Not only they are required to highly perform in the proximity of two electrochemical environments operating at two opposite extremes, but also provide acceptable bulk transport properties that allow them to respond swiftly to the power demands of the system. Additionally, it is essential that the electrolytes have acceptable safety properties which include high thermal stability, low volatility, low flammability, low toxicity, and low reactivity with ambient air. Therefore, developing electrolytes possessing the aforementioned traits, no doubt, represents a key challenge. Since the first rechargeable magnesium battery was demonstrated in the early nineties, the R&D efforts have primarily focused on the creation of electrolytes that are highly compatible with the magnesium metal, followed by applying innovative strategies to improve other electrochemical properties. A main focus was increasing their stability against electrochemical oxidation, so that a competitive, high voltage battery system could be ultimately enabled. Over the past two decades, the technical advancements made on magnesium battery electrolytes resulted in state of the art systems that primarily consist of organohalo-aluminate complexes possessing electrochemical properties that rival those observed in lithium ion batteries. These are represented by a highly reversible performance, high bulk conductivity, and wide electrochemical windows. However, despite these scientific feats, these electrolytes had several drawbacks which include their corrosive properties, nucleophilicity (for those Grignard based), air sensitivity and the use of volatile solvents.

Over the past two years, motivated by the desire to overcome the challenges with the known electrolytes, several new electrolytes that are compatible with the magnesium metal have been proposed. It is interesting to see that in previous and most recent electrolytes, the familiar monomeric and dimeric Mg–Cl active species were found. One important challenge with these same species is the slow transport properties. Another is the presence of chloride ions making them prime suspects in the corrosion issue. Hence, we believe that the discovery/design of new electroactive species is needed. Recent development in this direction is manifested in the borohydride electrolytes, where opportunities for increasing the oxidative stability are being explored and were demonstrated using *closo*-borane anions. Another common property among magnesium electrolytes is their air sensitivity. New approaches offered lowered sensitivity to air using alkoxides and phenolates. It would be interesting to determine their long term durability and see future designs that build on these systems, which are hopefully non-corrosive. In order to overcome the challenges with the liquid systems, solid electrolytes could be an ideal choice. The discovery of magnesium compatability and conduction in magnesium amide borohydride inspires confidence in this direction.

Indeed, the portfolio of magnesium battery electrolytes has widened and we hope that the current research will fuel the next wave of innovations. This could be driven by further understanding of the properties of the electrolytes and their behavior in a battery system. Topics we suggest include: 1) Discerning the electroactive species and their interactions with both the magnesium metal and the cathode material. This may prove powerful in paving the path for designing modified electrolytes; 2) Determining important electrochemical transport properties in both the bulk and at the interface with the magnesium metal; 3) Understanding the extent of the air stability, thermal stability and long term durability of the electrolyte; 4) Understanding the effects of a battery environment on the electrochemical stability window. For example, examining the oxidative stability on the cathode material rather than solely using metals/glassy carbon electrodes; 5) Lastly, developing corrosion resistant substrates, such as pretreated surfaces, as this may be helpful in overcoming the corrosion issue. However, we think that this effort may be worthwhile when electrolytes become demonstrated with very competitive performances.

Would future electrolytes help magnesium metal one day become the “ultimate battery anode”? There is no clear answer at this time. However, the numerous breakthroughs and scientific advancements made so far make one hopeful that at least it may have come one step closer.

### 3 Rechargeable Mg battery cathode

Much effort has been devoted to development of Mg batteries and their cathodes over the past 70 years. Some cathode materials have been practically investigated for a reserve-type Mg battery system, which were typically used together with Mg and Mg–Al–Zn (AZ) alloy as anode, and electrolytes based on either sea water or magnesium perchlorate (Mg(ClO_4_)_2_) solutions. The reserve battery requires high energy density, high power output, long lifetime and superior low temperature performance. Therefore, typical examples of cathodes for such Mg batteries, which have been summarized in the battery handbook so far were AgCl, CuCl, PdCl_2_, Cu_2_I_2_, CuSCN, MnO_2_ and air [1]. These batteries could be operated as primary batteries which fulfilled the aforementioned requirements, however they could not be operated as secondary batteries enabling us to recharge them. One of the reasons considered for the non-rechargeability was the water passivation of the anode surface. As the metal was exposed to water, a blocking layer such as Mg(OH)_2_ was formed accompanied with hydrogen gas generation. To recharge the battery, applying large overpotential was necessary due to the formation of highly resistive blocking layer, and finally the interface between anode and electrolyte, which determines the battery performances, could not be fully recovered. Due to the major hurdles with the anode, the challenges of Mg battery cathode may have been masked. Actually, a proper understanding of the cathode reaction and a further enhancement of the cathode performance are vital to realize the rechargeable system. Nowadays, the studies on rechargeable Mg battery cathodes have gained more momentum in order to improve the primary battery characteristics such as voltage, capacity, cycle life and so on. Currently, traditional cathode materials which are briefly summarized below have been intensively reconsidered recently, while other candidates, which are not described in this review, have been addressed. For example, Mg_0.5_Ti_2_(PO_4_)_3_ [55], Mg*_x_*MnSiO_4_ [56–58], WSe [59], sulfur [24,60] and oxygen [61–63]. In order to discover the next generation Mg battery cathode, the most important challenges are overcoming the negative impact arising from divalent Mg^2+^ ions and also maintaining higher mobility of Mg^2+^ ions in the diffusion pathway. So far, despite the research efforts to overcome these challenges, the very slow diffusion of Mg^2+^ ions and the structural instability remain as key hurdles in the development of working high voltage cathodes. Here, we strongly focus on the recent progresses of representative cathode materials for rechargeable Mg batteries.

#### 3.1 Cobalt-based cathode materials

**Figure 8:**
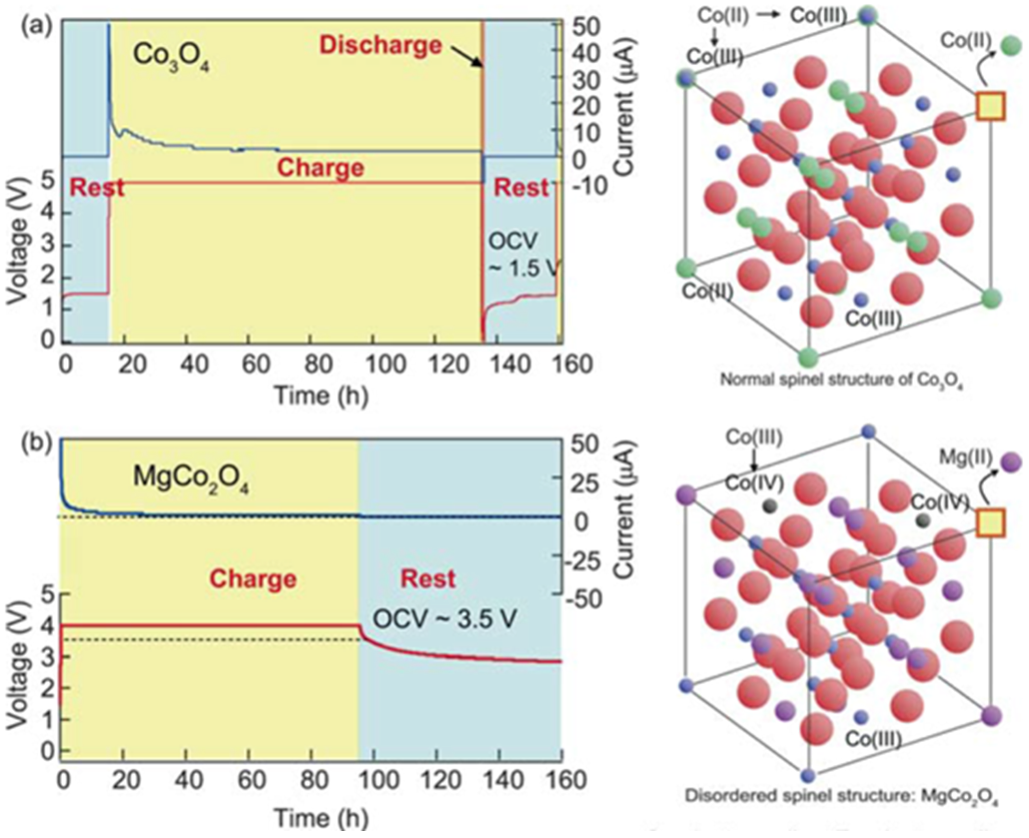
The OCV values of the test cells after constant voltage charge: positive electrode, (a) ordered Co_3_O_4_ and (b) disordered MgCo_2_O_4_; negative electrode, Mg; electrolyte, Mg(ClO_4_)_2_ in acetonitrile. Reproduced with permission from [66]. Copyright 2011 The Royal Society of Chemistry.

Since the 1990s, a variety of non-aqueous electrolytes have been adopted to evaluate and improve the rechargeability of the battery. It was believed that the non-aqueous electrolytes consisting of either magnesium perchlorate in acetonitrile solvent or magnesium organoborate in tetrahydrofuran solvent were capable of overcoming the issue resulting from water-containing systems. Gregory et al. surveyed some candidates among many cathode materials by chemical intercalation experiments using typical electrochemical methods [12]. Based on XPS analyses it was reported that ZrS_2_ as a host material was able to receive Mg. Also, they proposed that RuO_2_ and Co_3_O_4_ were hopeful candidates expected to capture Mg ions. These materials were also studied by Sutto et al. to demonstrate the redox capability in a different non-aqueous electrolyte system [64–65]. According to their discussion, Co_3_O_4_ did not allow for a sufficient magnesium insertion because of i) strong interactions between Mg^2+^ cations and oxygen atoms in the host lattice and ii) a drastic change of host structure and particle size after magnesiation. The initial capacity of 74 mAh g^−1^ was observed at around 1.5–2.0 V against Mg and a capacity retention of 60% after 30 cycles was reported. As such, the magnesiated Co_3_O_4_ did not show the high capacity obtained in the Li system. Recently, Ichitsubo et al. studied disordered MgCo_2_O_4_ (precisely, (Mg_1−_*_x_*Co*_x_*)(Mg_1−_*_y_*Co*_y_*)O_4_) which is a semiconductor with high electrical conductivity [66]. Compared with spinel-phase Co_3_O_4_, the disordered spinel, MgCo_2_O_4_ increased the open circuit voltage (OCV) by about 2.0 V at the initial stage just after a constant voltage charge as shown in Figure 8. One reason for this increase could be resulting from an enhanced Mg^2+^ ion diffusion compared to the ordered structure. The disordered spinel Mg_0.67_Ni_1.33_O_2_ also showed high OCV based on the same principle [66]. Unfortunately, the high initial voltage (over 3.0 V vs Mg) observed for these spinel materials could not be maintained during the rest time following charging as a continuous voltage decay was observed. This meant that these cathode materials possessed high polarization due to slow diffusion of Mg^2+^ in the host lattice. Thus, even for these materials it was not possible to discharge the battery at a higher voltage over 3.0 V vs Mg. Although these cathodes did not enable stable high voltage performance, the introduction of the disordered structure into the Mg battery cathode is indeed a good idea to neutralize the local charge density occurring between inserted Mg^2+^ ions and the host lattice, and to furthermore accelerate intrinsic Mg^2+^ ion diffusion.

#### 3.2 Vanadium-based cathodes

**Figure 9:**
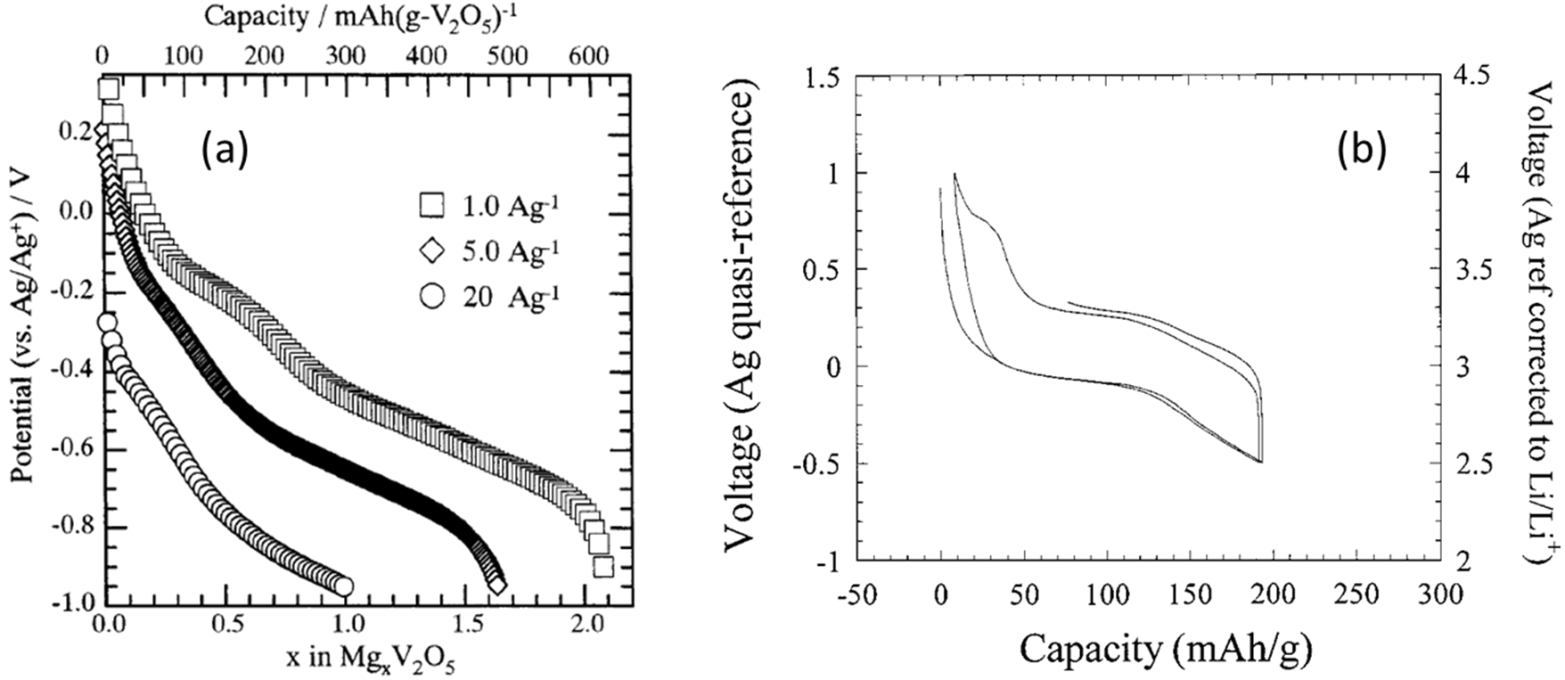
(a) Discharge curves of V_2_O_5_/carbon composites in the Mg(ClO_4_)_2_/acetonitrile electrolyte solution at various current densities. Reproduced with permission from [70]. Copyright 2003 ECS–The Electrochemical Society. (b) Charge–discharge curves of nanocrystalline V_2_O_5_ in the Mg(ClO_4_)_2_/propylene carbonate electrolyte solution. Reproduced with permission from [72]. Copyright 2001 ECS–The Electrochemical Society.

Other well-researched materials for Mg battery cathodes are V_2_O_5_ and a series of vanadate oxides [67–72]. Novak et al. proposed a water-containing V_2_O_5_ cathode system in an organic electrolyte such as Mg(ClO_4_)_2_ in propylene carbonate [68–69]. It was expected that V_2_O_5_ was capable of accommodating 2 mol of Mg^2+^ ions, which is equivalent to the V^5+^/V^3+^ redox reaction. However, according to their report, the electrochemical insertion of Mg^2+^ ion into V_2_O_5_ was dependent on the amount of water in the electrolyte. Additionally, the maximum content of Mg^2+^ ions was observed to be less than 0.6 mol. This is because chemically bounded water was present in the channel of the V_2_O_5_ host which prevented further magnesiation. Although the observed capacity was much lower than the expected value, hydration of Mg^2+^ ions is expected to mitigate difficulty of their electrochemical insertion into the host lattice as explained in a previous review [69]. Imamura et al. followed this approach and used V_2_O_5_ xerogel and its carbon composite, where the V_2_O_5_ xerogel with 50 nm in thickness was coated on the carbon support [70–71] (Figure 9a). It was reported that in the wide operation range, the xerogel enabled achieving high content of magnesiation of up to *x* = 1.84 in Mg*_x_*V_2_O_5_, resulting in a high capacity of about 600 mAh g^−1^ in the potential range from −1.0 to +0.3 V vs Ag/Ag^+^. On the other hand, Amatucci et al. demonstrated the electrochemical performance of V_2_O_5_ in the non-aqueous media with negligible amount of water [72] (Figure 9b). Nano-sized V_2_O_5_, which had a particle size distribution of 20–50 nm, brought about a higher discharge capacity and a narrower hysteresis with a higher working voltage than micron-sized V_2_O_5_. In both Imamura’s and Amatucci’s approaches, using a thin layer and nanoparticles allowed for a short diffusion length of Mg^2+^ ions, thereby improving the Mg battery performance. Although the possibility of H_2_O associated intercalation for V_2_O_5_ has only been suggested, these approaches could be eventually one of the important ways that help accelerate Mg^2+^ ion diffusion in the lattice.

#### 3.3 Molybdenum-based cathodes

**Figure 10:**
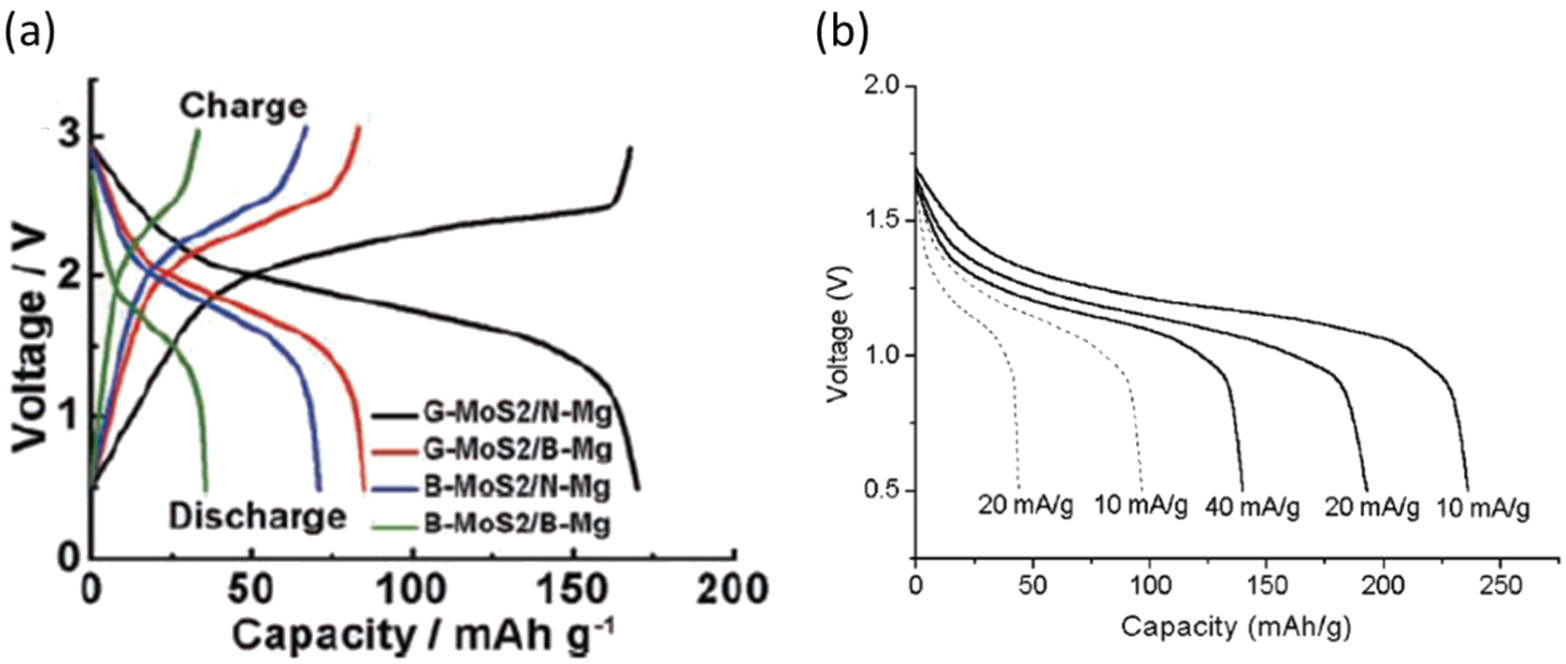
(a) Charge–discharge curves of graphene-like MoS_2_ in the Mg(AlCl_3_Bu)_2_/tetrahydrofuran electrolyte solution. Reprinted with permission from [74]. Copyright 2011 Wiley-VCH Verlag GmbH & Co. KGaA, Weinheim. (b) Discharge curves of TiS_2_ nanotubes in the Mg(ClO_4_)_2_/acetonitrile electrolyte solution at various current densities. Reproduced with permission from [77]. Copyright 2004 The Royal Society of Chemistry.

A big success in developing a cathode for Mg batteries was presented in 2000 by Aurbach et al. They discovered an excellent material, the Chevrel phase (CP) Mo_6_S_8_, as a rechargeable Mg battery cathode [20]. The CP cathode was proven to have a very stable performance with less than 15% capacity fade over 2000 cycles at 100% depth of discharge. Note that practical rates of 0.1–1.0 mA/cm^2^ and wide temperature ranges from −20 to +80 °C were used. As described in previous articles [7,20,73], these promising properties are enabled by the following features of the CP cathode; 1) electroneutrality derived from delocalized Mo_6_ metallic cluster, 2) plenty of sites per cluster where Mg^2+^ ions can be accommodated for solid-state diffusion, 3) high electronic conductivity. One of the drawbacks of the CP cathode is that the kinetics of Mg^2+^ ion diffusion was strongly dependent on the composition and operating temperature [23]. During initial magnesiation, 20–25% of Mg^2+^ ions were trapped in the CP lattice and were not extracted unless the temperature was elevated. Moreover, when the CP cathode was tested at a low temperature around 15 °C, the capacity of about 80 mAh g^−1^ observed at 1/10 C rate, decreased to about 40 mAh g^−1^ at 1 C rate. An effective countermeasure to promote fast kinetics of the CP cathode was the partial substitution of Mo_6_S_8_ by Se. It was observed that the Se-substituted CP cathode showed an excellent accessibility of Mg^2+^ ions, resulting in a higher capacity at higher rate and at lower temperature. Unfortunately, the CP cathode families showed relatively low working voltages at around 1.2 V vs Mg and relatively low capacities of around 110 mAh g^−1^. To make the magnesium battery more practical, a Mg cathode with high energy density is strongly desired. Recently, it was reported that graphene-like MoS_2_ also worked as a Mg battery cathode [74–76] (Figure 10a). Chen et al. found that this material exhibited an operating voltage of 1.8 V and a reversible capacity of about 170 mAh g^−1^ by combining with Mg nanoparticles as an anode. Additionally, in a similar way, TiS_2_ has been considered as a common cathode material even in Mg batteries [77] (Figure 10b). The operating voltage of TiS_2_ was not high enough compared with Mo_6_S_8_, and it suffered from limited rate and temperature performances. However, a higher capacity of about 180 mAh g^−1^ vs Mg was obtained by the state of art nanotechnology. Therefore, transition metal sulfides as prototypical intercalation host materials may bring in a new breakthrough for Mg battery cathodes.

#### 3.4 Manganese-based cathodes

**Figure 11:**
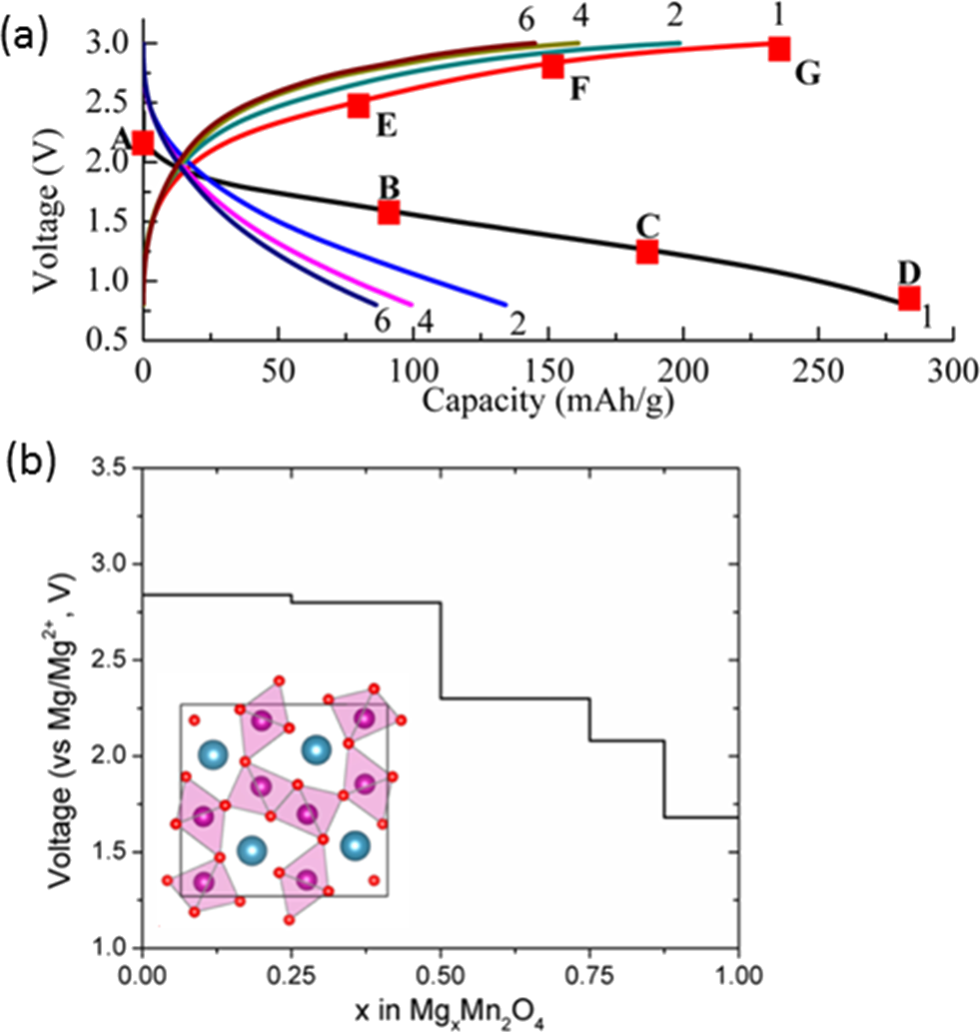
(a) Charge–discharge curves of α-MnO_2_ in organohaloaluminate/tetrahydrofuran electrolyte solution. Reprinted with permission from [81]. Copyright 2012 Elsevier. (b) Predicted voltage for electrochemical insertion of Mg into the post spinel, MgMn_2_O_4_ which was drawn in the inset. Adopted with permission from [82]. Copyright 2013 American Chemical Society.

Finally, the remaining attractive materials as Mg battery cathode was MnO_2_ and its polymorph [78–82]. MnO_2_ is widely regarded as a common cathode material in primary batteries including either Zn or Mg anodes, in lithium-ion secondary batteries and furthermore in metal–air batteries. The unique MnO_2_ polymorphs have been used as Mg battery cathodes coupled with either a magnesium organohaloaluminate electrolyte solution or magnesium perchlorate non-aqueous electrolyte solution. In 2011, Zhang et al. presented the redox capability of α-MnO_2_ during magnesiation and demagnesiation [81] (Figure 11a). α-MnO_2_ with 2×2 tunnel structure showed a high initial capacity of about 240 mAh g^−1^ and could be repeatedly discharged and recharged. Unfortunately, this cathode had severe capacity fading due to a drastic structural deformation from the tetragonal phase to the orthorhombic phase during magnesiation. While this is known to occur in all manganese-based cathodes for lithium-ion batteries, such structural instability during magnesiation is thought to be a key trigger that severely deteriorates α-MnO_2_ in non-aqueous Mg batteries. Very recently, Ling et al. proposed an alternative manganese material for a Mg battery cathode, which was called a post-spinel compound, MgMn_2_O_4_ with 2×2×1×1 structure [82] (Figure 11b). Theoretical calculations predicted that the above-described structural stability was significantly improved by controlling the tunnel size and shape for Mg^2+^ ion diffusion. As a result, the post-spinel compound facilitated Mg insertion/extraction reaction more than α-MnO_2_ with bigger tunnel size, and then had a relatively high operation voltage. In addition, the cooperative hopping of Mg^2+^ ions in the tunnel 2×2×1×1 was estimated to aid faster diffusion, resulting in a low diffusion barrier (≈400 meV) that is comparable with that for LiMn_2_O_4_, a typical lithium-ion battery cathode. Thus, the structural modification for Mg^2+^ ion diffusion is also one of the approaches which could be used to achieve fast kinetics in the cathode and minimize the interactions of strongly bounded Mg^2+^ ions with host tunnels.

#### 3.5 Perspectives on rechargeable cathodes

In order to establish the non-aqueous Mg battery as a system, it is noteworthy that the cathode strongly governs the battery performance. High energy density of the cathode is an indispensable requirement for Mg batteries to become a reality. To realize this, two approaches can be generally followed; one is having a high voltage operation, while the other is having a high capacity operation. In the latter, despite the fact that either sulfur or oxygen cathodes have to be truly demonstrated for Mg battery, they may offer potential high capacity future cathodes. However, it is expected that these cathodes would have challenges similar to those encountered in the Li battery system. An important point would be whether the typical issues present in the Li–air and Li–sulfur systems could be solved using the Mg system. Herein, we focus on the cathode materials for high voltage operation. Generally, oxide-based materials should be suitable to meet such a request, since oxide-based materials are theoretically able to show higher redox potential as demonstrated in lithium-ion batteries. However, in terms of the Mg^2+^ ion mobility, oxide materials currently have the issue of sluggish diffusion. This resulted in an overall battery performance that was not so promising compared to that using the sulfide materials.

To the best of our knowledge, there have been a couple of key solutions to overcome this undesirable situation. First is to discover an appropriate host structure with faster kinetics as was already discussed above. From the viewpoint of the guest ion size relative to the host structure, Mg^2+^ as a guest ion does not have an issue because the ionic radius of Mg^2+^ (0.74 Å) is close to that of Li^+^ (0.68 Å). However, when considering the interaction between the guest ion and the host structure, the divalent nature of Mg^2+^ ions notably suppresses fast diffusion observed in the monovalent Li^+^ system, because of the: 1) tightly bounded attraction between Mg^2+^ and the host and 2) strong repulsion between Mg^2+^ ions. As a result, sluggish diffusion of Mg^2+^ ions causes poor magnesiation and non-dynamic situation which means that mobile ions are stuck either in the diffusion pathway or on the surface. Structural designs that promote Mg^2+^ ion diffusion is thought to be the best way to discover promising Mg battery cathodes operating at high voltage.

Another key challenge is how to control the charge transfer resistance observed at the cathode/electrolyte interface, which should become more apparent after the sluggish diffusion issue is fully overcome. In fact, the charge transfer resistance has been carefully studied in lithium-ion batteries and was found to have a significant role that determines the battery performance. A surface film formed on a cathode active material should allow for transporting Mg^2+^ ions, but sometimes it may act as a blocking layer, thus hampering the charge transport. Even though the solvated Mg^2+^ ions can go through the surface film, a desolvation process needs to take place before the ions could migrate inside a host structure. Probably, Mg^2+^ ions would have a stronger solvation than Li^+^ ions, therefore the charge transfer resistance is expected to be considerably higher. In a practical setup, this is an important factor necessary to promote further magnesiation. As it has been the case for state of art technologies such as those in lithium-ion batteries, Mg battery electrolytes will also need to be optimized for such high voltage operation of the cathode. The cathode/electrolyte interface will have to be considered so as we do not lose the superior electrochemical properties especially those for the high voltage cathodes.

Finally, for high voltage battery operation, close attention needs to be paid to the corrosion of the current collectors. In any environment having an electrolyte salt (e.g., magnesium perchlorate and magnesium organoborate) dissolved in an organic solvent, the corrosion of current collectors must be suppressed in order to properly monitor the cathode properties in a battery setup. In particular, high voltage systems need to be understood in a suitable electrolyte environment with wide electrochemical window. Further progresses in Mg battery cathodes are needed and should go hand-in-hand with the developments of non-corrosive and electrochemically stable electrolytes.

## Conclusion

Indeed, current state of the art rechargeable magnesium battery technologies are far from reaching its promised potential, where several hurdles, particularly resulting from the absence of appropriate electrolytes and high capacity/voltage robust cathodes remain. Nonetheless, we are hopeful that an improved understanding of the chemistry/physics of these batteries and future innovative ideas may after all allow for battery engineering and system optimization per application needs. This may enable commercialization of these batteries, sooner or later.
